# Mitochondrial quality control in hepatic ischemia-reperfusion injury^☆^

**DOI:** 10.1016/j.heliyon.2023.e17702

**Published:** 2023-06-27

**Authors:** LiuSong Wang, Zan Jie Feng, Xuan Ma, Kai Li, Xin Yao Li, Yi Tang, Cijun Peng

**Affiliations:** a Department of General Surgery, Affiliated Hospital of Zunyi Medical University, Zunyi, China; b Department of Biochemistry and Molecular Biology, Zunyi Medical University, Zunyi, China

**Keywords:** Hepatic ischemia-reperfusion injury, Mitochondrial quality control mechanisms, Mitophagy, Mitochondrial dynamics, Mitochondrial biogenesis

## Abstract

Hepatic ischemia-reperfusion injury is a phenomenon in which exacerbating damage of liver cells due to restoration of blood flow following ischemia during liver surgery, especially those involving liver transplantation. Mitochondria, the energy-producing organelles, are crucial for cell survival and apoptosis and have evolved a range of quality control mechanisms to maintain homeostasis in the mitochondrial network in response to various stress conditions. Hepatic ischemia-reperfusion leads to disruption of mitochondrial quality control mechanisms, as evidenced by reduced mitochondrial autophagy, excessive division, reduced fusion, and inhibition of biogenesis. This leads to dysfunction of the mitochondrial network. The accumulation of damaged mitochondria ultimately results in apoptosis of hepatocytes due to the release of apoptotic proteins like cytochrome C. This worsens hepatic ischemia-reperfusion injury. Currently, hepatic ischemia-reperfusion injury protection is being studied using different approaches such as drug pretreatment, stem cells and exosomes, genetic interventions, and mechanical reperfusion, all aimed at targeting mitochondrial quality control mechanisms. This paper aims to provide direction for future research on combating HIRI by reviewing the latest studies that focus on targeting mitochondrial quality control mechanisms.

## 1 Introduction

Hepatic ischemia-reperfusion injury (HIRI) is a pathological phenomenon in which the blood flow supplying the liver is temporarily blocked during hepatic surgery, and the damage to liver tissue is exacerbated when the liver's blood supply is restored. HIRI may result from liver transplantation, haemorrhagic shock, trauma, and hepatectomy for benign and malignant tumours [1]. In recent years, epidemiological studies have revealed a gradual increase in the incidence of liver advanced cancer, and liver transplantation is the main modality for tumours with intermediate to advanced liver cancer [2]. The presence of HIRI significantly lowers the success rate of liver transplants and increases the risk of complications for patients undergoing this procedure [3,4]. The pathological mechanisms underlying HIRI are complex, is not fully understood yet, mainly including oxidative stress, aseptic inflammation, Ca^2+^ overload, and apoptosis [5].

Mitochondria are double-membrane organelles, Mitochondria play a major role in glucose metabolism, inflammation, reactive oxygen species (ROS) signalling calcium homeostasis and cell death [6]. Mitochondrial dysfunction as the pathological mechanism of human related aging and a variety of diseases [7,8]. Under stress conditions, such as ischemia-reperfusion, mitochondrial damage or dysfunction leads to reduced ATP synthesis, causing an overload of calcium ions due to limited ATP-dependent ion transport processes. Damaged mitochondria generate ROS via the electron transfer chain, causing oxidative harm to mitochondrial components such as phospholipids, proteins, and DNA [9]. As a result, healthy mitochondria lose their functionality [10]. Calcium overload and ROS trigger mitochondrial MPT, leading to the release of harmful substances that worsen organ damage through the activation of apoptosis and inflammatory responses [11]. In normal situations, the body maintains the health of its mitochondria through quality control mechanisms. These mechanisms include autophagy, fusion, fission, and biogenesis, which help to remove dysfunctional mitochondria and renew mitochondrial components quickly [12,13]. However, during organ ischemia-reperfusion, these mechanisms are disrupted. This causes poor mitochondrial autophagy, fragmentation, and reduced biogenesis. In turn, this results in cell apoptosis through the mitochondrial pathway, ultimately worsening the damage caused by the ischemia-reperfusion injury [14]. One of the important pathological mechanisms of HIRI [15]. Therefore, it is a breakthrough to prevent and cure hepatic ischemia reperfusion injury by improving mitochondrial injury and restoring the function of mitochondrial network.

Although the pathological mechanism of mitochondrial quality control disorders is unclear, many scholars have reported that pharmacological approaches improve mitochondrial function by regulating mitochondrial quality control and alleviating ischemia reperfusion injury [15,16]. In this paper, with mitochondrial quality control as the core. We summarized various interventions for preventing and treating HIRI in recent years, focusing on mitochondrial autophagy, kinetics, and biogenesis. Our aim is to provide guidance for further research towards preventing and treating HIRI, while also establishing a solid theoretical foundation for future strategies.

## 2 Mechanism of HIRI-induced mitochondrial apoptosis

The mechanisms underlying hepatic ischemia-reperfusion injury can be categorized into two distinct stages: ischemic injury and reperfusion injury. During ischemia, anaerobic glycolysis replaces aerobic respiration, limiting ATP and antioxidant production. This results in the accumulation of acidic metabolites and cytoplasmic acidosis due to the hydrolysis of ATP and production of H^+^[17,18]. The cytoplasmic acidosis has a protective effect on cells by inhibiting the function of enzymes, such as proteases and phosphatases, due to the acidic environment. Additionally, it inhibits the opening of the MPT, but can promote its opening during the reperfusion period after restoring a normal pH level [19]. The depletion of ATP also limits the ion transport process, the reduced calcium pump (Ca2+-ATPase pumps) activity inhibits endoplasmic reticulum calcium reuptake and mediates cytoplasmic calcium overload. Furthermore, a malfunctioning Na + -K + -ATPase pump disrupts the equilibrium of sodium-potassium exchange, causing increased cytoplasmic permeability, inward water movement, and subsequent cell swelling [18]. Studies have shown that cytosolic Ca2+ triggers the opening of mitochondrial MPT, causing the release of ions, metabolic intermediates, and inducing permeability conversion. This leads to depolarization of the mitochondrial membrane potential, uncoupling of oxidative phosphorylation, and mitochondrial swelling, ultimately resulting in apoptosis via the mitochondrial pathway [11]. In the physiological state, ROS production is maintained at baseline levels and is cleared by antioxidant enzymes. During reperfusion, the Xanthine oxidase, NADPH oxidase, mitochondrial electron transport chain, and uncoupled nitric oxide synthase (NOS) systems use O2 as an electron acceptor. This leads to the production and release of superoxide (O2•-) and hydrogen peroxide (H_2_O_2_), which results in increased oxidative stress [20]. Positive feedback damage to mitochondria due to oxidative stress occurs through sustained production of ROS, which stimulates the opening of the mitochondrial MPT, to induce cells apoptosis [21]. Studies show that, a small molecule compound, DS44170716, also defends human liver HepG2 cells against Ca2+ -induced death by blocking the opening of MPT [22]. Treatment of cyclosporin A specifically blocks onset of the MPT in rats alleviated hepatic apoptosis and inflammatory response mediated by hemorrhagic shock [23]. Mitochondrial quality control mechanisms are crucial for maintaining the homeostasis of the network in a physiological state [12,13]. Impaired mitochondrial quality control can lead to damaged mitochondria that trigger persistent apoptosis and inflammation, thus worsening hepatic ischemia-reperfusion injury [[24], [25], [26]].

## 3 Mitochondrial biogenesis in HIRI

Mitochondrial biogenesis involves the proliferation and division of extant mitochondria. It involves a series of processes involving both mitochondrial DNA and nuclear DNA, including the transcription and translation of mitochondrial respiratory chain-related enzymes, membrane phospholipid synthesis, and mitochondrial DNA replication.

The transcriptional coactivator peroxisome proliferator-activated receptor α (PPARα) coactivator 1α (PGC-1α), as a major regulator of mitochondrial biogenesis, is localized in the nucleus and discovered by Spiegelman and colleagues in 1998 [27]. PGC-1α is crucial in promoting mitochondrial biogenesis by acting as a coactivator for other mitochondrial transcription factors. It further encourages the expression of NRF-1 and NRF-2α mRNA [28]. Nuclear respiratory factor 1/2 (NRF-1/2), are important participants to enhance transcription of encode subunits of the five key respiratory complexes, in addition, interaction between PGC-1 and NRF-1, and then binds to the promoter region of mitochondrial transcription factor A (TFAM) to promote transcription, which is responsible for transcription and replication of mtDNA [29,30].

**Fig. 1:**
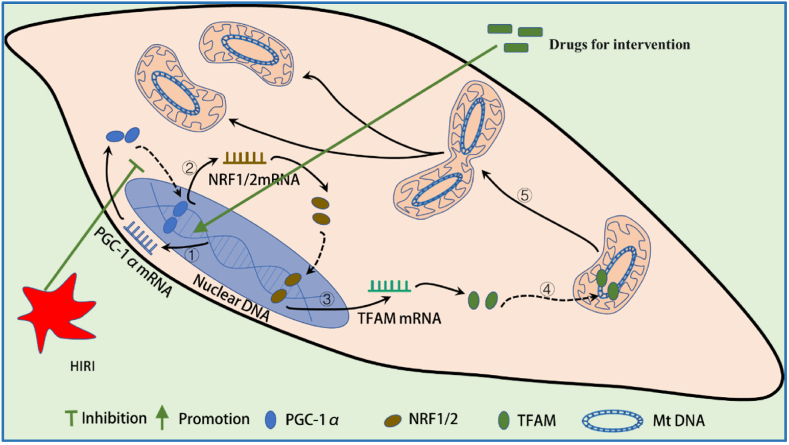
The process of mitochondrial biogenesis and the mechanisms of therapeutic agents which protect against HIRI by promoting mitochondrial biogenesis. ①: therapeutic agents promoting the expression of the transcriptional coactivator peroxisome proliferator-activated receptor α (PPARα) coactivator 1α (PGC-1α) mRNA, a common transcriptional regulator of others transcription factors. ②：PGC-1α promotes the expression of Nuclear respiratory factor 1/2(NRF1/2) by binding to the promoter region of the NRF1/2 gene, which promoting the transcription and translation of enzymes associated with the mitochondrial respiratory chain, acts as transcription factors. ③: NRF1/2 also promotes the expression of mitochondrial transcription factor A (TFAM). ④: TFMA protein facilitates mitochondrial DNA replication. ⑤:Eventually inducing mitochondrial biogenesis, mitochondrial division leads to increased functional mitochondria.

Given the important role of PGC-1α in mitochondrial biogenesis, its dysfunction is involved in the development and progression of various mitochondria-related diseases. These include Parkinson's disease [31], cancer [32], and diabetes [33]. However, mitochondrial biogenesis is significantly inhibited during I/R induction in liver tissue. In the in vivo and in vitro models of HIRI, the processes of ischemia and reperfusion, which inhibit the expression of PGC-1α, NRF-1, NRF-2, and TFAM, lead to a decrease in mtDNA copy number and ATP content and an increase ROS production. In addition, pro-apoptotic factors are released into the cytoplasm, inducing hepatocyte apoptosis [34,35]. Studies have shown that treatment with herbal monomers, biotin, or with technical manipulations can increase the transcription and translation of genes associated with mitochondrial biogenesis through the upregulation of PGC-1α expression, promoting the restoration of the mitochondrial network and attenuating apoptosis in liver tissue (Table 1), (Fig. 1). Exogenous irisin and exosomes from adipose-derived mesenchymal stem cells (ADSCs‐exo) markedly inhibits the expression of the mitochondrial fission-related proteins Drp-1 and Fis-1 and increases expression levels of PGC-1α and TFAM, thus increasing the mitochondrial content in a rat model of hepatic I/R [34,35]. Research by Shin showed that Genipin significantly improved hepatic I/R injury, increased hepatic ATP levels and increasing mtDNA copy number by partially promoting mitochondrial biogenesis via increasing the expression of PGC-1α, NRF-1, and TFAM [15]. In addition, Normothermic machine perfusion (NMP) also significantly upregulated the expression of TFAM [36]. Therefore, in treating HIRI, targeting PGC-1α can promote mitochondrial biogenesis, increase the number of mitochondria, and improve mitochondrial function, all of which will help to inhibit apoptosis and protect against HIRI.

**Table 1:** Summary of strategies to reduce hepatic I/R injury by regulating mitochondrial dynamics and biogenesis. ADSC-exo: exosomes derived from adipose-derived mesenchymal stem cells; OPA1: optic atrophy 1; PGAM5: phosphoglycerate mutase 5; CDK1: cyclin-dependent kinase 1; Drp1: dynamin-related protein 1; YY1/UBA2: transcription factor Yin Yang-1/a subunit of SUMO-E1 enzyme heterodimer; Mfn2: mitofusin1/2; PGC-1α: transcriptional coactivator peroxisome proliferator-activated receptor (PPARα) coactivator 1α; TLR4: Toll-like receptor 4; MIC60: subunit mitochondrial contact site and cristae organising system.

Intervention means	mechanism of action	reference
exogenous irisin	mitochondrial fission/biogenesis	[34]
Genipin	mitochondrial fission/fusion biogenesis	[15]
Treprostinil	mitochondrial fission/fusion	[37]
Nobiletin	mitochondrial fission/fusion; autophagy; biogenesis	[38]
Polyethylene glycol (PEG35)	mitochondrial fission/fusion; autophagy, biogenesis	[39]
Cyclooxygenase 2 (COX-2)	OPA1-dependent mitochondrial respiration	[40]
Haem oxygenase-1 (HO-1)	PGAM5–dependent mitochondrial fission; mitochondrial biogenesis, mitophagy	[41]
Normothermic machine perfusion (NMP)	mitochondrial fission; biogenesis	[36]
Hepatic stimulator substance (HSS)	mitochondrial fission	[42]
Augmenter of liver regeneration protein (ALR)	mitochondrial fission	[43]
Drp1 inhibitor Mdivi-1; calcineurin FK506	mitochondrial fission	[44]
Breviscapine	mitochondrial function by upregulation Mfn2	[45]
Unacylated ghrelin	mitochondrial function activated Opa1/MIC60	[46]

## 4 Mitochondrial dynamics in HIRI

Mitochondria are dynamic organelles that regulate energy metabolism, and their morphology and distribution are controlled through fission and fusion. Together these processes are known as mitochondrial dynamics. Mitochondrial fission is mainly mediated by DRP1 (dynamin-related protein 1), which is predominantly located in the cytoplasm. After undergoing phosphorylation, it becomes activated and actively migrates to the surface of mitochondrial membrane. There, it binds with the receptor FIS1 and collaborates with the mitochondrial fission factor (Mff) and mitochondrial elongation factor 1 to form a splitting complex that triggers the contraction of mitochondria and cleavage of membranous structures [47,48]. Has attracted attention because DRP1-mediated mitochondrial fission is closely associated with the induction of apoptosis. DRP1 activity is mainly regulated by phosphorylation modifications, with phosphorylation of two different Ser sites exerting opposite regulatory effects on DRP1 activity: phosphorylation of Ser616 enhances the activity of Drp1 and phosphorylation of Ser637 inhibits it. The final activity depends on the ratio of phosphorylation at these two sites [49]. In addition to phosphorylation, small ubiquitin-like modification (SUMOylation) also enhances Drp1-mediated fission activity of mitochondrial [43]. Multiple phosphatases and kinases regulate DRP1 activity, linking various cellular events to mitochondrial fission [50].

The merging of two mitochondria into one is known as mitochondrial fusion. The fusion is mediated by mitofusin1/2 (Mfn1/2), which is located in the outer mitochondrial membrane, and optic atrophy 1 (OPA1), which is located in the intra- and intermembrane spaces [12]. Two adjacent mitochondria are tethered through a dimeric antiparallel coiled-coil structure in Mfn1/2 [51] and form the Mfn-OPA1 protein complex, which initiates the fusion of the inner and outer membranes [52]. Eventually the mitochondria will become elongated. Fusion of mitochondria with a stable membrane potential and isolation of depolarized mitochondria is essential for the maintenance of mitochondrial homeostasis [53].

**Fig. 2:**
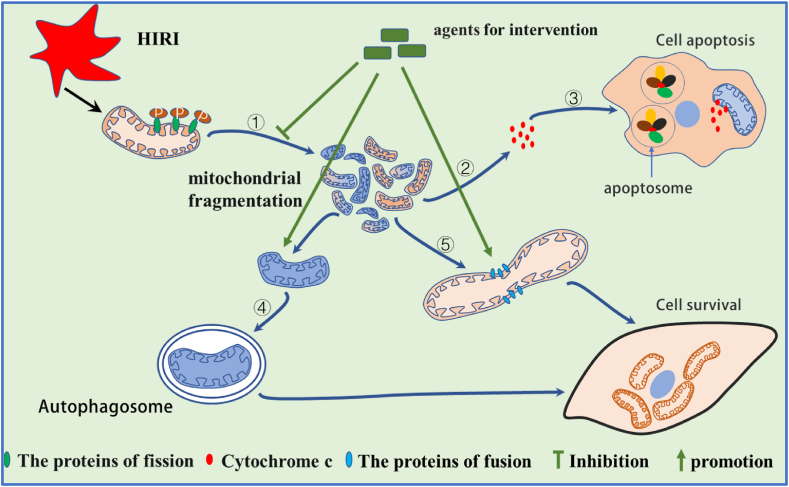
Therapeutic agents protect the liver against ischemia-reperfusion injury by promoting mitochondrial fusion, mitophagy and inhibiting fission. ①:HIRI triggers fragmentation of mitochondria by promoting the phosphorylation of DRP1; ②:This fragmentation of mitochondrial structure promotes the release of cytochrome C into the cytoplasm; ③:Cytochrome C, in turn, activates apoptotic vesicles, leading to programmed cell death; ④:Certain drugs remove depolarized mitochondria by promoting mitochondrial autophagy; ⑤:Other drugs improve mitochondrial function by facilitating mitochondrial fusion.

DRP1-mediated fission is necessary for brain development, but when it exceeds the normal level and fusion is hampered, it results in mitochondrial fragmentation, which is indicative of apoptosis. This condition is common in a range of diseases, such as hepatic ischemia-reperfusion injury [54,55]. Various substances enhance HIRI by controlling DRP1 phosphorylation and preventing its translocation and fission in the mitochondria. Mice haploinsufficient for hepatic stimulator substance (HSS), a liver growth-promoting factor (HSS^+/−^), demonstrates deteriorated liver I/R injury. In vitro studies found that HSS overexpression suppresses Drp1 translocation to mitochondrial, which reduces the release of cytochrome *c* by inhibiting CDK1/cyclin B-dependent phosphorylation of Drp1 Ser616 [42]. In HEK293T and HepG2 cell, ALR overexpression significantly inhibits Drp1 SUMOylation by interacting with Yin Yang-1 (YY1)/*UBA2* pathway, and attenuating IRI-induced mitochondrial fission and preserving mitochondrial function [43]. In mouse hippocampal neurons, HIRI induced the expression of calcineurin, which dephosphorylation at the Drp1 Ser637 and promoted the translocation of DRP1 to mitochondria, leading to mitochondrial fragmentation and cell apoptosis [44]. In contrast, the Drp1 inhibitor Mdivi-1 and the calcineurin inhibitor FK506 both up-regulated phosphorylation levels at the Drp1 Ser637 site, thus improving hippocampal neuronal apoptosis [44]. Developing targeted inhibitors or activators for phosphatases and phosphorylation sites that regulate DRP1 activity in HIRI is a promising approach. Recent studies have shown that not only do phosphorylation processes regulate DRP1 activity, but multiple measures can also decrease DRP1 expression, prevent apoptosis, and improve the damage caused by HIRI. Pharmacological approaches, stem cells, mechanical perfusion [36], etc. Are reported, (Table 1), (Fig. 2). Pharmacological approaches include: **natural plant active ingredients**: such as Genipin [15]; Breviscapine, a traditional Chinese medicine and a flavonoid derived from the natural plant *Erigeron breviscapus* [45]; nobiletin, a polymethoxyflavone primarily present in citrus fruits [38];**Human synthetic biotin**, such as irisin is a secreted fragment of fibronectin type III domain containing 5 (FNDC5) [34]; cyclooxygenase 2 (COX-2), a key enzyme in prostanoid biosynthesis [40]; haem oxygenase-1 (HO-1), an endogenous cytoprotective enzyme [41]; unacylated-ghrelin (UnAG), a form of ghrelin that is mainly produced in the stomach [46];**Stem cells and exosomes**, such as exosomes derived from adipose‐derived mesenchymal stem cells (ADSCs‐exo) [35]. Furthermore，Treprostinil is an FDA-approved PGI2 analogue with potent vasodilatory and anti-platelet aggregation properties. Recent studies have found that Treprostinil improve rat liver injury induced by renal I/R injury via regulating mitochondrial dynamics and restore mitochondrial function [37]. Polyethylene glycol (PEG35) are safe and neutral compounds. They have been approved by the FDA for use in cosmetics, foods and drugs，a study show that PEG35 preconditioning improves mitochondrial dysfunction by promoting mitochondrial fusion and inhibiting mitochondrial fission in vitro model of hypoxia/reoxygenation [39]. Therefore, Treprostinil and PEG35 are promising drugs for the prevention and treatment of HIRI, but there is a lack of corresponding clinical trials. A systematic review revealed that Mdivi-1，the Drp1 inhibitor， as a potential therapy to reduce brain damage in ischemic stroke [56]. However, it has not yet been studied in HIRI. Due to technological limitations and potential toxic side effects of drugs, translating basic research to the clinical setting is hindered. Even though biotin and stem cells are safe, technical constraints currently limit their use in clinical settings. However, exploring their potential for drug development holds promise for the future.

### 4.1 Mitochondrial autophagy in HIRI

Autophagy, involves degradation of misfolded protein, damaged and surplus organelles, and lipid droplets, and invasive exotic organisms [57], is a critical survival response to starvation [58]. Mitochondrial autophagy, also known as mitophagy, involves the selective removal of damaged or non-functional mitochondria and is an integral part of mitochondrial quality control. Defective mitophagy contributes to the progression of a large number of ageing-related diseases, such as cancer, Alzheimer disease, and hepatic insulin resistance [[59], [60], [61]].

The process of HIRI includes ischaemic and reperfusion phases. During the ischaemic phase, the sudden cessation of blood supply causes cellular hypoxia, activating intracellular autophagic signals to reduce unnecessary consumption of limited energy for cell survive. However, in cases of prolonged ischemia, ATP is almost completely depleted, which eventually inhibits autophagy since it is a process that consumes a lot of energy [62]. In addition, ATP depletion severely limits the transport of Ca^2+^ by the highly ATP-dependent Ca^2+^ pump and other ion exchangers in the cell membrane, resulting in Ca^2+^ overload. Ca^2+^overload activates calpain, thereby promoting the degradation of autophagy-associated proteins such as ATG7, Beclin-1, and ATG4B, leading to the formation and prolongation of autophagic vesicles and the reduction of autophagic flux [63,64]. During the reperfusion stage, despite restoring oxygen supply, mitochondria are temporarily repolarised, which promotes ATP production, further inducing autophagy and facilitating the clearance of dysfunctional mitochondria. However, the introduction of oxygen also stimulates the production of mitochondrial reactive oxygen species (ROS) [65,66], leading to extensive mitochondrial damage through a feed-forward mechanism. The threshold for mitophagic flux to clear damaged mitochondria was exceeded. On the one hand, calcium ion overload and ROS release trigger mitochondrial permeability transition (MPT), which enables small molecules (less than 1500 Kda) to diffuse through the mitochondrial inner membrane into the cytoplasm. This activates an apoptosis cascade resulting in cell apoptosis [67,68]. On the other hand, the accumulation of damaged mitochondria in the cytoplasm acts as a risk factor, activating a sterile inflammatory response by releasing mitochondrial contents such as mitochondrial DNA, residual ATP, and mitochondrial proteins. These substances are recognised as danger-associated molecular patterns (DAMPs) [69,70], ultimately resulting in tissue damage.

Therefore, enhancing mitochondrial autophagy may reduce HIRI. However, instead of the continued increase observed in young mice, in old mice, the level of LC3B declined 60 min after reperfusion. In the livers of old mice subjected to I/R, increased cell apoptosis and mitochondrial ROS production was observed compared with that in the younger group. Pre-treatment with the autophagy inhibitor chloroquine (CQ) in the liver of young mice subjected to I/R blocked mitophagy flux, which increased the levels of the apoptosis of hepatocytes than in the control group [24].

**Fig. 3:**
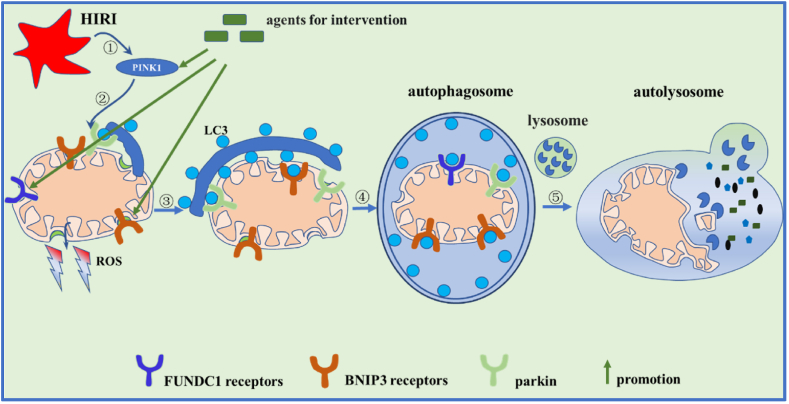
**Pharmacological intervention protects against hepatic ischemia-reperfusion injury by promoting mitophagy.** ①:Drugs enhance PINK1 expression; ②:PINK1 facilitates parkin movement to mitochondria; ③: parkin initiates mitophagy by ubiquitinating mitochondrial outer membrane proteins; ④: parkin, FUNDC1, and BNIP3 associate with LC3 to form autophagosomes around damaged mitochondria; ⑤:Autophagosomes merge with lysosomes, and acidic enzymes degrade mitochondria. (For interpretation of the references to colour in this figure legend, the reader is referred to the Web version of this article.)

Recent studies have found that activation of protective mitophagy is mediated in two main ways. One，**PINK1/Parkin-mediated mitophagy**, two, **Receptor-mediated mitophagy. PINK1/Parkin-mediated mitophagy**: PINK1/Parkin-dependent autophagy is relatively widely studied in disease, while knowledge about receptor-mediated autophagy is lacking. Briefly, mitophagy mediated by tensin homologue-induced putative kinase 1 (PINK1). During physiological conditions, PINK1 is rapidly degraded by mitochondrial membrane peptidases and (PARL) presenilin associated, rhomboid-like (PARL) protein in mitochondrial matrix, and the cleaved fragments of PINK1 are delivered into the cytoplasm, where it binds to Parkin and inhibits mitochondrial translocation of Parkin, and mitophagy is inhibited [71,72]. When mitochondria encounter stress and damage, PINK1 cannot translocate and is degraded by PARL due to membrane potential depolarization. PINK1 accumulates on depolarized mitochondria's outer membrane and activates Parkin by phosphorylation, starting mitophagy [73,74]. Parkin is an E3 ubiquitin ligase that marks multiple outer mitochondrial membrane proteins with ubiquitin [75]. Parkin's ubiquitin ligase activity targets MFN1/2 and voltage-dependent anion channel (VDAC), leading to their degradation via LC3 interaction and ultimately triggering damaged mitochondrial autophagy. Recent studies have found that pharmacological preconditioning, ischemic preconditioning [76], and mechanical reperfusion can all improve HIRI to some extent in animal and cell models by activating PINK1/Parkin-mediated mitophagy (Fig. 3). The drugs used for pretreatment mainly include **active ingredients extracted from plants**, such as Genipin, the active ingredient extracted from Gardenia fruits [15]; a specialised pro-resolving lipid mediator, Resolvin D1 (RvD1) [16]; pterostilbene (Pt), an active ingredient in berries and grapes [77]; **Biotin synthesized by the human body**, such as 25-hydroxycholesterol (25 H C), a proxy for oxysterol; Augmenter of liver regeneration (ALR) is an anti-apoptotic protein found mainly in mitochondria [78]. Haem oxygenase-1 (HO-1), an endogenous cytoprotective enzyme [41]; **Stem Cells** [79]; **Synthetic molecular activators and inhibitors**, such as SRT1720, a small molecule allosteric stimulator of Sirtuin 1 (Sirt1) [80], salubrinal, an inhibitor of the protein phosphatase PP1 [24];**Gene interventio**n：such as: DJ-1 [81]，PINK1 [82]，parkin [83,84]. They all demonstrated that powerful protection effect on hepatic I/R injury by activating PINK1/parkin-dependent mitophagy in animal model.

Thus far, the discussion of mitophagy in HIRI has centred on the PINK1/Parkin pathway. Furthermore, dysfunctional mitochondria can also express autophagy receptors to activate mitophagy. Hypoxia induces the expression of mitophagy-related receptors that contain interaction sequences with LC3. By playing a bridging role and binding to both cargo molecules and LC3, these mitophagy receptors facilitate the autophagic elimination of damaged mitochondria [57]. These receptors mainly include BNIP3/NIX and FUNDC1. **BNIP3/NIX**: a B-cell CLL/lymphoma 2 (BCL-2)-related protein, playing a dual role in inducing apoptosis and mitophagy. Phosphorylation of Ser17 and Ser24 on BNIP3 LIR motif positively regulates its binding to LC3, promoting pro-survival mitophagy instead of apoptosis in mammalian cells [85]. **FUNDC1**, an outer mitochondrial membrane protein, plays an essential role in hypoxia-induced autophagy. Its autophagic activity is regulated through dephosphorylation and phosphorylation**.** Under hypoxia condition, dephosphorylation of FUNDC1 at serine 13 (Ser-13) by mitochondrial phosphatase phosphoglycerate mutase family member 5 (PGAM5) and phosphorylation at Ser17 b y Unc-51 like autophagy activating kinase 1 (ULK1), which ultimately induces mitophagy [86,87]. FUNDC1 is phosphorylated on Tyr18 b y the Src kinase reduces its binding with LC3 to inhibit mitophagy [86]. Furthermore, damaged mitochondria release cardiolipin to its surface, which then binds to LC3 to activate mitophagy [88]. To develop targeted drugs against hepatic ischemia-reperfusion injury, it is important to identify upstream regulatory kinases or phosphatases that control BNIP3 and FUNDC1, and investigate their relationships with other molecules.

Studies support that activating mitophagy by promoting relevant receptors protects against ischemia-reperfusion injury. In models of myocardial and renal I/R injury, HIF-1α/BNIP3 has been found to promote mitophagy, inhibiting apoptosis and serving as a cell survival pathway [89,90]. In vivo and in vitro I/R injury model, deletion of Mst1 recovered FUNDC1 expression, thus re-activating protective mitophagy and blocking mitochondrial dependent apoptosis [91]. Bnip3 knockout enhanced apoptosis and inflammatory response in kidneys in renal tubular epithelial cells oxygen-glucose deprivation-reperfusion (OGD-R) and in renal tubules after renal I/R injury in mice [92]. Promoting FUNDC1-dependent mitophagy by Empagliflozin protected cell apoptosis against myocardial I/R injury [93]. Tissue-type fibrinogen activator (tPA) is a thrombolytic agent used to medically treat ischemic stroke. Recent studies show that it can prevent cerebral neuronal apoptosis caused by I/R by promoting autophagy through FUNDC1 [94]. Electroacupuncture (EA) and Trimetazidin are approved for treating ischemic reperfusion injury in the heart and brain. Studies suggest that EA's protection stems from regulating mitochondrial autophagy [95,96]. Their safety has been proven in clinical practice, so could they be considered for the prevention and treatment of mitochondrial damage in HIRI? Few studies have examined FUNDC1 and BNIP3-mediated mitochondrial autophagy in HIRI, and the limited research available reveals histological disparities in the function of BNIP3. Specifically, compared to findings in I/R injury of other organs. BNIP3 appears to exacerbate HIRI [97]. Propofol (PRO) protects against hepatic I/R injury by reduced cell apoptosis via inhibiting Bnip3 [98]. Understanding how receptors regulate mitochondrial autophagy in hepatic ischemia-reperfusion injury is crucial in complementing the complex pathological mechanisms of HIRI.

In addition, mitophagy is regulated by other signalling pathways, such as：Sirt1-Mnf2 axis, Sirtuin 3 (Sirt3)-mediated mitophagy, DRP1/Beclin-1 signalling, PINK1/Parkin-dependent autophagy is also regulated by AMPK. Activation of these pathways-mediated mitochondrial autophagy protects against hepatic ischemia-reperfusion injury [99,100]. Activating Sirt1-mediated mitophagy signalling using methoxyestradiol (2-ME2) protects I/R-induced hepatocellular from damage in alcoholic fatty liver (AFL) [101]. Enhanced mitophagy and alleviated cell death in vivo and in vitro after hepatic I/R through activating dynamin-related protein 1 (Drp1)/Beclin-1 signalling by CHOP depletion [102]. AMPK also plays an important role in the activation of mitophagy [79]. In liver I/R injury in diabetic mice, activation of AMPK effectively restored mitophagy activation, leading to decreased mitochondrial oxidative stress and attenuated liver I/R injury [103]. This implies that mitochondrial autophagy is governed by other proteins within the mitochondria, creating a complex regulatory network. Additionally, there is mutual control between mitochondrial dynamics and autophagy. As a result, there is much more to comprehend regarding the regulatory mechanisms of mitophagy.

## 5 Conclusion and future directions

According to the theory of bacterial evolution, mitochondria evolved from bacteria capable of aerobic respiration and evolved to play a crucial role in cell survival and death. Over time, mitochondria have evolved a quality control mechanism to maintain mitochondrial homeostasis. Mitochondrial quality control mechanisms include biogenesis, kinetics, and mitochondrial autophagy. To maintain a healthy mitochondrial network, three mechanisms should operate in tandem: fusing functional mitochondria with stable membrane potential, splitting and eliminating damaged ones through fission and mitophagy, and providing synthetic feedstock for biogenesis. The disturbance of mitochondrial quality control mechanisms can cause an imbalance in mitochondrial network homeostasis. This can be observed through excessive fission, suppressed mitophagy, and inhibited mitochondrial renewal, which results in the persistence of damaged mitochondria. In turn, this induces a local inflammatory response and apoptosis. Evidence shows that damaged mitochondria aid in their own clearance by undergoing ubiquitin degradation of MNF1/2 during autophagy. This leads to a loss of fusion capacity and prevents damaged mitochondria from rejoining the healthy mitochondrial network. This highlights the connection between autophagy and mitochondrial dynamics. The potential interplay between mitochondrial autophagy, kinetics, and biogenesis remains under-reported, making it crucial to uncover their regulatory networks in order to comprehensively grasp the significance of mitochondrial quality control in disease prevention and management. Recent studies have uncovered additional mitochondrial proteins, including mitochondrial deacetylase 1/3, the fusion protein mfn1/2 and the fission protein DRP1, that are crucial in mediating mitochondrial autophagy alongside PINK1/Parkin-dependent mitophagy and receptor-mediated autophagy. These proteins form intricate regulatory pathways, and comprehending their functional roles and regulatory mechanisms serves as a valuable complement to the mitochondrial autophagy mechanism. This provides a theoretical foundation for targeted drug development against HIRI.

Various substances and methods have been discovered to alleviate HIRI in basic experiments. Unfortunately, none of these drugs have been approved for clinical trials, partly because certain natural plant components are limited in use due to their toxicity and complex extraction process. Although human synthetic biotin and stem cells, as well as their exosomes, are non-toxic, they have been limited to animal experiments and have not yet been translated to clinical use due to immature extraction techniques and low yields. The next research goals should focus on developing non-toxic plant active ingredient derivatives and advancing extraction technology. With these advancements, it is expected that significant progress will be made toward preventing and treating HIRI in the near future.

## Author contribution statement

All authors listed have significantly contributed to the development and the writing of this article.

## Data availability statement

No data was used for the research described in the article.

## Funding statement

This study was supported by the 10.13039/501100001809Natural Science Foundation of China [81660688].

Guizhou Postgraduate Research Fund Project (Qianjiaohe YJSKYJJ (2021) 176).

## Declaration of competing interest

The authors declare that they have no known competing financial interests or personal relationships that could have appeared to influence the work reported in this paper.
